# On-site electronic consent in pediatrics using generic Informed Consent Service (gICS): Creating a specialized setup and collecting consent data

**DOI:** 10.1371/journal.pdig.0000661

**Published:** 2024-11-25

**Authors:** Katharina Danhauser, Larissa Dorothea Lina Mantoan, Jule Marie Dittmer, Simon Leutner, Stephan Endres, Karla Strniscak, Jenny Pfropfreis, Martin Bialke, Dana Stahl, Bernadette Anna Frey, Selina Sophie Gläser, Laura Aurica Ritter, Felix Linhardt, Bärbel Maag, Georgia Donata Emily Miebach, Mirjam Schäfer, Christoph Klein, Ludwig Christian Hinske

**Affiliations:** 1 Department of Pediatrics, Dr. von Hauner Children’s Hospital, University Hospital, LMU Munich, Munich, Germany; 2 German Center for Child and Adolescent Health, Munich Site; 3 Medical Technology and IT (MIT), University Hospital, LMU Munich, Munich, Germany; 4 Institute for Community Medicine, Department Epidemiology of Health Care and Community Health, University Medicine Greifswald, Greifswald, Germany; 5 Trusted Third Party of the University Medicine Greifswald, Greifswald, Germany; 6 Institute for Digital Medicine, University Hospital Augsburg, Augsburg, Germany; Beth Israel Deaconess Medical Center, UNITED STATES OF AMERICA

## Abstract

Enrolling in a clinical trial or study requires informed consent. Furthermore, it is crucial to ensure proper consent when storing samples in biobanks for future research, as these samples may be used in studies beyond their initial purpose. For pediatric studies, consent must be obtained from both the child and their legal guardians, requiring the recording of multiple consents at once. Electronic consent has become more popular recently due to its ability to prevent errors and simplify the documentation of multiple consents. However, integrating consent capture into existing study software structures remains a challenge. This report evaluates the usability of the generic Informed Consent Service (gICS) of the University Medicine Greifswald (UMG) for obtaining electronic consent in pediatric studies. The setup was designed to integrate seamlessly with the current infrastructure and meet the specific needs of a multi-user, multi-study environment. The study was conducted in a pediatric research setting, where additional informed consent was obtained separately for the biobank. Over a period of 54 weeks, 1061 children and adolescents aged 3 to 17 years participated in the study. Out of these, 348 agreed also to participate in the biobank. The analysis included a total of 2066 consents and assents, with 945 paper-based and 1121 electronic consents. The study assessed the error susceptibility of electronic versus paper-based consents and found a significant reduction rate of errors of 94.7%. These findings provide valuable insights into the use of gICS in various studies and the practical implementation of electronic consent software in pediatric medicine.

## Author summary

As researchers we need to make sure that people who participate in a clinical trial, study or biobank give their informed consent before they take part. This is especially important when samples are stored for future research, as they may be used in ways beyond their original purpose. In pediatric studies, we need to obtain consent from both the child and their legal guardians, which requires recording multiple consents at once. Recently, electronic consent has become popular because it reduces errors and simplifies documentation. However, integrating this technology into existing study systems remains a challenge. Our work tested an electronic consent system called gICS, the generic Informed Consent Service of the University Medicine Greifswald, in a pediatric research setting. We wanted to see how well it worked for obtaining multiple consents from children and adolescents aged 3 to 17 years. Over 54 weeks, we collected more than 2000 consents using both paper and electronic methods. Our results show that most errors in paper-based consents were due to missing signature dates. Electronic consent significantly reduced errors by almost 95%. The results provide useful information on how to use the gICS in different studies and how to implement electronic consent software in pediatric healthcare.

## Introduction

Informed consent is a critical component of good clinical practice (GCP) when conducting clinical trials and studies. Its primary objective is to safeguard study participants and ensure that their participation in a clinical study is voluntary and informed [1]. In accordance with EU Regulation (EU) 2016/679, commonly referred to as the General Data Protection Regulation (GDPR), obtaining informed consent from the data subject, their legal representatives, or guardians (Articles 6–9 GDPR) is generally necessary for the collection, processing, and use of medical research data [2]. Consent is typically acquired through a paper-based form that is signed by the subject, and their legal guardians, or legal representatives. However, this method lacks structured and machine-readable data, which can make efficient storage and retrieval problematic. To make consent forms available electronically, they often need to be manually entered into a software system, a process that can be time-consuming and error-prone. A structured process is important for easily locating specific decisions made by a subject [3].

Risks and challenges of electronic consent have been analyzed, including data protection and privacy standards [4]. The need to ensure compliance with regulations and to achieve interoperability of electronic systems is of critical importance [5,6]. Although there may be some drawbacks such as data protection concerns or usability and accessibility challenges, the advantages of electronic consent are undeniable. Studies have shown that electronic consent significantly reduces error rates. For instance, research on medical consent has demonstrated that paper-based handwritten forms can produce an error rate of up to 50%, caused by errors in the documentation of procedures details, the absence of signatures and the absence of dates. The aforementioned error rate was significantly reduced by using electronic consent [7–9]. Electronic consent forms also reduce the administrative burden on study staff, freeing up more time for interaction with study participants. Standardization, the generation of comprehensive data, and increased efficiency and effectiveness offer opportunities that were previously unavailable [10].

The acquisition of electronic consent in pediatrics has not been extensively studied. The available information typically pertains to the digital tools used in research to describe the procedures, rather than the process of obtaining consent [10]. Obtaining consent for pediatric studies is guided by specific regulations to protect the rights of children. Generally, child’s parents or legal representatives must provide consent for their enrollment in a study. In some cases, depending on the child’s age and maturity, the child’s assent may also be required [11]. Therefore, two consents are obtained for a study: one from the parents or legal representatives and the other from the child, depending on their age and decisional capacity [11–13]. In this publication, the term ’consent’ refers to the consent of both the legal representatives and the child. As biobanks are increasingly used in academic research to store biological samples for future studies, it may be necessary to obtain additional informed consent from participants. For this reason, in pediatric studies, up to four consents may be collected from a subject as part of the consent process.

This study aims to evaluate the effectiveness of the generic Informed Consent Service (gICS) as a software solution in the consent process for research, particularly in multi-user and multi-study settings. gICS was developed by the Institute for Community Medicine (ICM) at the University Medicine of Greifswald. It manages consent templates, which are stored in a structured, versioned format based on modules and assigned policies. These consent templates might be visualized directly on a tablet to document the participant’s will [14].

## Materials and methods

### Software and technical setup

**Fig 1:**
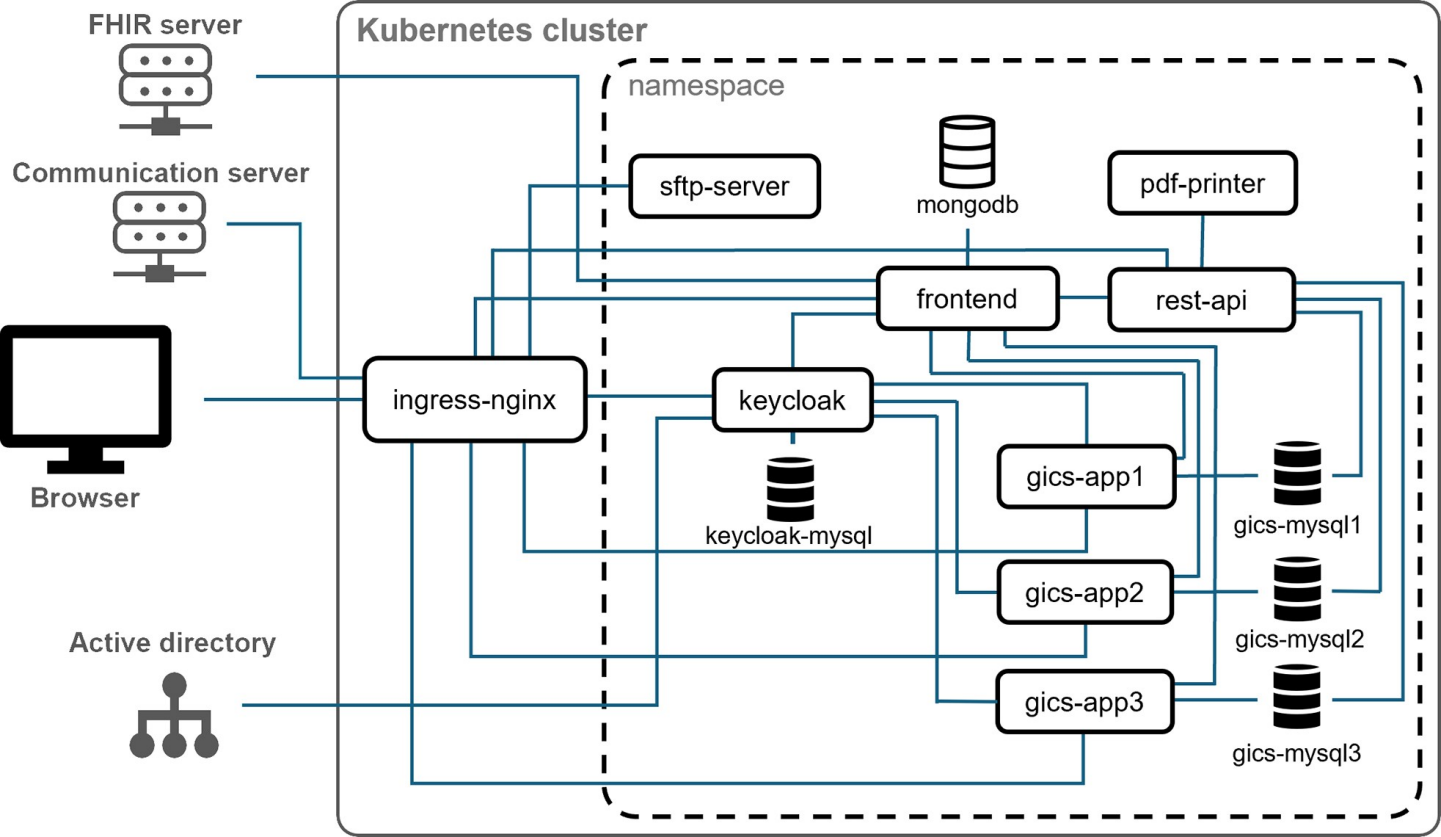
Schematic representation of the overall setup. Arrows have been omitted for better clarity. The applications are accessible through the browser. Authentication and authorization are handled through Keycloak, which has its own database (keycloak-mysql) and is connected to the Active Directory. The requests are forwarded to the applications via ingress-nginx. The frontend allows for the entry or retrieval of respondent information, such as the participant’s name, via a FHIR server. The consent templates are selected and a personalized QR code is generated for each consent. These templates are retrieved from the gICS and prefilled with the necessary information, ensuring that they are ready when the QR code for the subject information is accessed. The subject’s completed consent is temporarily stored in the mongodb database and transferred to gICS after final signature by the person providing information. This example displays various instances of gICS, including gics-app1, gics-app2, and gics-app3. The templates and consents are stored in the corresponding databases of the gICS, namely gics-mysql1, gics-mysql2, and gics-mysql3. The REST interface (rest-api) can be used to poll consents incrementally. The PDF printer (pdf-printer) performs regular queries and automatically generates and stores a PDF of the consent. The PDF can then be accessed through the SFTP server (sftp-server). The communication server retrieves the consent data from the gICS databases and transfers the PDFs along with the consent details to other relevant systems.

The open-source software gICS, was utilized to create consent forms and record electronic consents. At the beginning of the evaluation, the most recent version available to start with was 2.15.2, which has since been updated to version 2024.1.1. gICS is available in a containerized format (Docker, https://docker.com), allowing for virtualization of software and an alternative method of running applications. Each container includes all the required components for the application to function independently of the underlying system’s specific configurations. During setup, we chose to exclusively use containers with the following adaptations (Fig 1).

Multi-tenancy: Access to the software must be granted through personal access rights assigned on a role-based basis for each study according to their respective tasks, ensuring compliance with data protection laws. This means that only authorized users may access the personal information provided in a consent. Since study-specific access was not available in the software up to version 2.15.2, a separate application was launched for each study, including a database. This setup ensures multi-tenancy by keeping the data strictly separate in different databases.

Authentication and authorization: Two authentication options are available in the software. These are internal user management called gRAS or the implementation of Keycloak, an open-source identity and access management software based on OpenID-Connect (https://www.keycloak.org). To integrate gICS into the existing user administration, a Keycloak was set up for a streamlined connection to the Active Directory. This also enabled the implementation of a single sign-on for various gICS instances and access control based on the current user roles and permissions.

Retrieval of consent content and automatic generation of signed consent documents: The setup includes an additional REST interface for retrieving consent templates and their content. This interface enables incremental retrieval of recently added consents from all databases. Furthermore, a PDF printer has been included in the setup to automatically generate PDF documents that contain consent template data and content. These documents can be accessed via an SFTP server within the setup.

**Fig 2:**
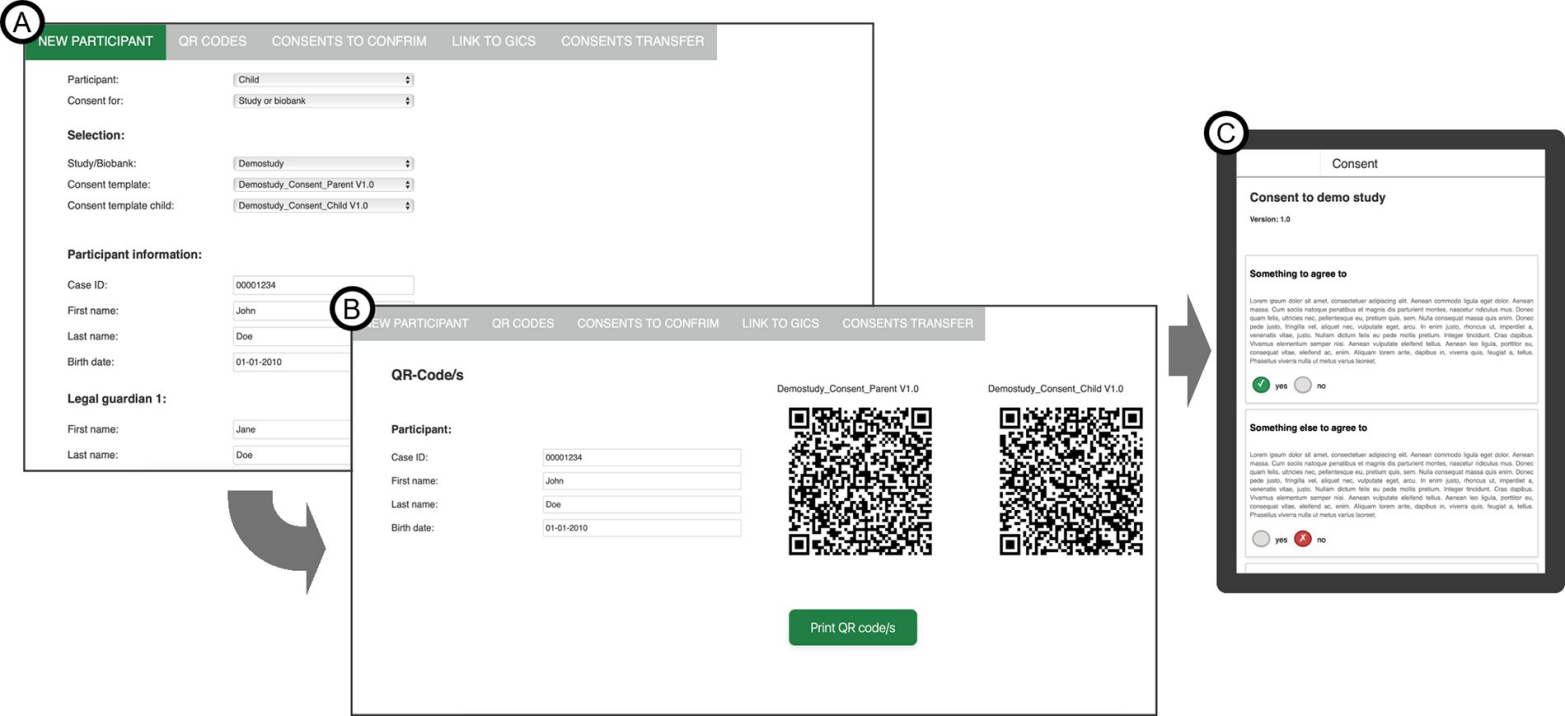
Screenshots of frontend’s user interface. Shown is the user interface for subject data entry (A) and QR code generation (B) of the application. After scanning the QR code, the participant can access and complete the consent form on a tablet (C).

Prefilling electronic consent forms with participant information: The gICS system permits the selection of a consent template. However, but it does not provide the capability to prepopulate fields such as participant names or identifications numbers within electronic forms. This limitation necessitated the development of a novel additional frontend, designed to facilitate personalization during electronic consent collection (Fig 2). By eliminating the need for redundant data entry across multiple consent forms for individual participants, this approach streamlined the consenting process. Through the user interface, study stuff can directly input participant information or retrieve it via a Fast Healthcare Interoperability Resources (FHIR) server using an identification number. Upon selecting a study and/or biobank, the corresponding consent template is accessed through the FHIR interface integrated within gICS [6]. The frontend API generates a personalized QR code for each consent. Participants can scan the codes to access, complete, review, sign, and temporarily save the prefilled electronic consent form after a brief login. Once the information provider signs the consent form, it is transmitted to gICS via the FHIR interface using the addConsent option.

Container management: The gICS setup was migrated to a Kubernetes cluster to simplify container management and automate the deployment of additional instances. Kubernetes is a container management software that coordinates cluster operations, including starting and stopping containers, distributing resources, and monitoring application health to ensure seamless and reliable performance [15].

### Study subjects

Electronic consent was obtained as part of a clinical study and enrollment of subjects in the pediatric hospital biobank with a group of pediatric participants aged 3 to 17 years. The study included patients from a range of pediatric subdisciplines, as well as healthy controls. The exclusion criteria for the study include lack of consent from the child or parents and lack of cooperation from the child. Ethics votes from the LMU Munich Ethics Committee were obtained for clinical study (No. 20–172), biobank (No. 20–111) as well as for the use of the software (No. 22–0225). For each participant, informed written consent was obtained from the legal representative/s.

### Creation of consent templates in gICS

gICS provides the option of storing consents in a modular format. The text of each component of the consent form is stored separately in modules, with policies designated for sections containing consent points. The template for the consent can then be constructed from the modules to yield the final template [14]. After converting the consent process of an ongoing study to a digital format, the paper-based consent templates were deposited in gICS as modules with corresponding policies for each consent point. This included parents’ consent forms and child’s assent forms, resulting in a total of four different templates.

### Informing process about the study and biobank

The initial contact with potential participants, including parents and children, was conducted by the study physicians. During the information phase, parents and children were given a comprehensive overview of the study’s objectives and procedures. Moreover, participants and their legal guardians were furnished with comprehensive information regarding the biobank and the storage and utilization of their biological specimens. Subsequently, parents and children were provided with detailed information documents regarding the study and the biobank, including the consent forms. To ensure that younger participants had a comprehensive understanding of the study and its implications, information documents were developed in a format tailored to the cognitive abilities of three distinct age groups [16–18]: 6–9 years, 10–13 years, and 14 years and above.

It is noteworthy that the information process was identical for both electronic and paper-based consent capture, thereby ensuring that all participants received the same level of information and had equal opportunities to ask questions.

### Collection of consents

At the time of recruitment, both the child and their parents or guardians were informed and educated about the study and the biobanking process, whereby consent to biobanking was not a prerequisite for participation in the study. Due to the wealth of information, consent forms were provided in advance to give parents and children sufficient time to familiarize themselves with the study and the biobank and allow them to make an informed decision regarding participation. Similarly, child’s assent was obtained from children and adolescents starting at the age of six years. The capacity to consent for children and adolescents was based on the assessment of parents and physicians. As the ability to sign was a prerequisite for consent, it was dependent on the age and health status of the child. The consent form was provided in paper-based form, printed from gICS, and was left unfilled. The content of the paper-based and electronic versions was identical, allowing for direct comparison. Informed consent forms for both the participating child and their legal representatives were verified or obtained during the final study appointment. Three scenarios were possible: 1) participants brought completed and signed paper-based consent forms, 2) participants brought some of the consent forms completed in paper form, or 3) participants did not bring any prefilled consent forms.

Accordingly, consents were captured using a hybrid approach combining paper-based and electronic versions. In the first scenario, paper-based consent forms were scanned, and content of the consent was documented in the study software. In the second scenario, all pending documents could be captured electronically, whereas prefilled documents that were brought on-site were paper-based. In the third scenario, electronic documentation was provided. In the latter two scenarios, parents and children were given the option to complete the consent forms on site, either in paper form or electronically. The electronically stored information and the generated PDF were stored in the study software. After completion and signature, the paper-based parts of all documents were scanned and stored in the study software. Furthermore, the consent contents were also entered into the study software.

The option to withdraw from participation in the study or biobank was available at any time. To guarantee the most straightforward withdrawal process possible, no written revocation was necessary. Consequently, withdrawals were accepted in a variety of formats, including verbally and in any other form of communication, and documented directly in the study software by the study staff.

### Error frequency analysis

As part of a regular quality check, paper-based documents were reviewed for errors and incorrect information using a checklist. Any necessary documentation was completed in the event of inaccuracies. According to general guidelines consents have to be signed and dated [2,14]. In addition, the consents have been checked for completeness and accuracy. Following potential errors were included in regularly quality checks: missing signatures of participants and/or persons providing information, missing date of signatures of participants and/or persons providing information, missing selection of consent options, misspelling of the participant’s name, additional text on the consent form, crossing out paragraphs on the form and incorrect completion of information on the consent form. Electronic consents were also checked for possible errors after collection. Number of errors in the paper-based and electronic consents was compared using a one-sided Z-test and the relative error reduction calculated.

## Results

### Age and sex distribution of study subjects

**Fig 3:**
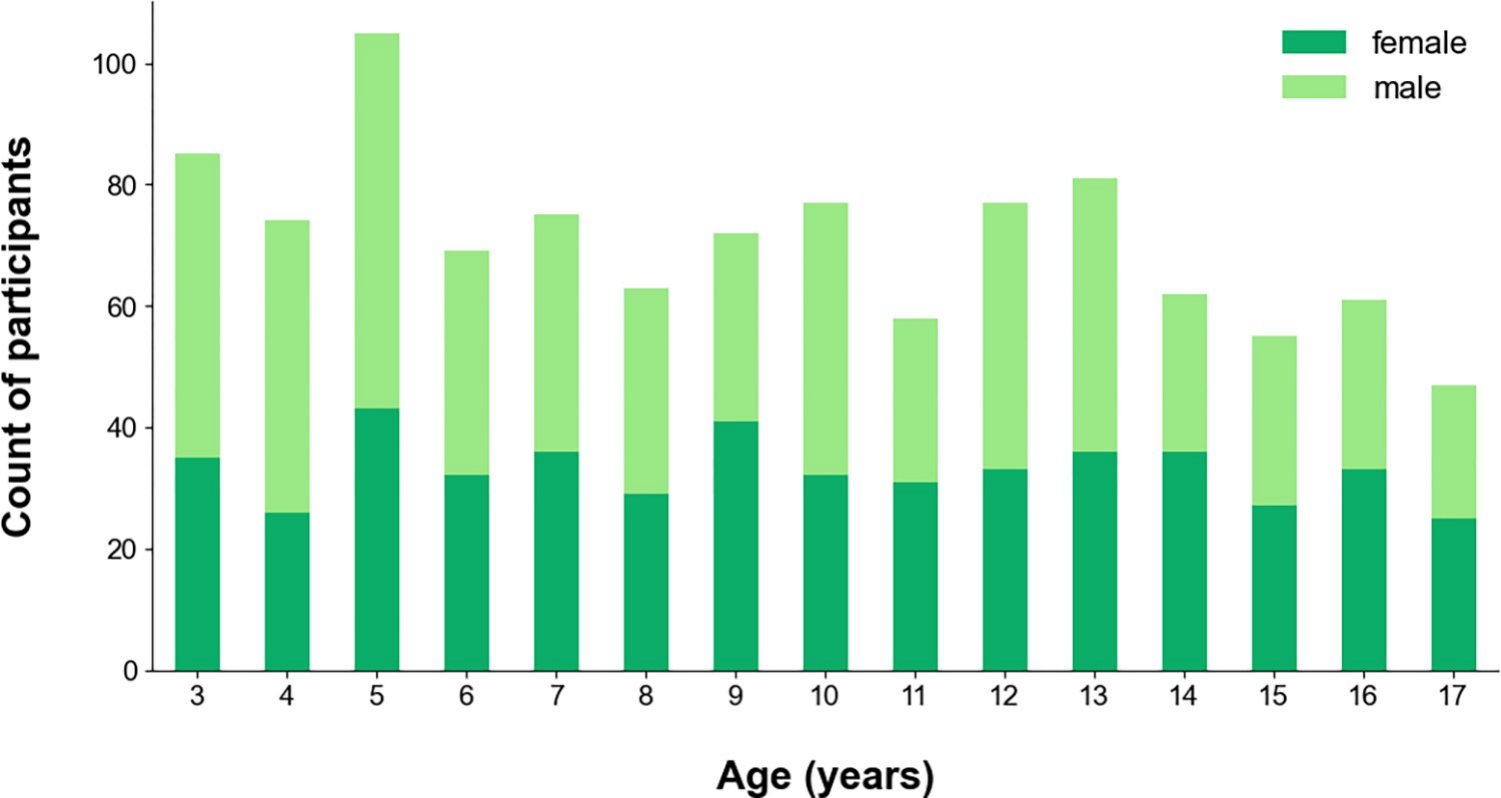
Age and sex distribution of participants. 495 female and 566 male participants aged 3 to 17 years took part.

A total of 1061 children and adolescents aged 3 to 17 years were included over 54 weeks. There was a good balance between the different age groups (Fig 3). The mean age was 9.5±4.3.

### Integration in existing infrastructure

The implementation into the current systems has proceeded smoothly, allowing for seamless integration. The REST interface, which has been additionally incorporated into the setup, can be used to gradually retrieve consent content from all databases, enabling its transfer to other systems through a communication server. Additionally, the PDFs can be transmitted to an archiving system via the SFTP server established within the setup.

### Process of taking consents

**Fig 4:**
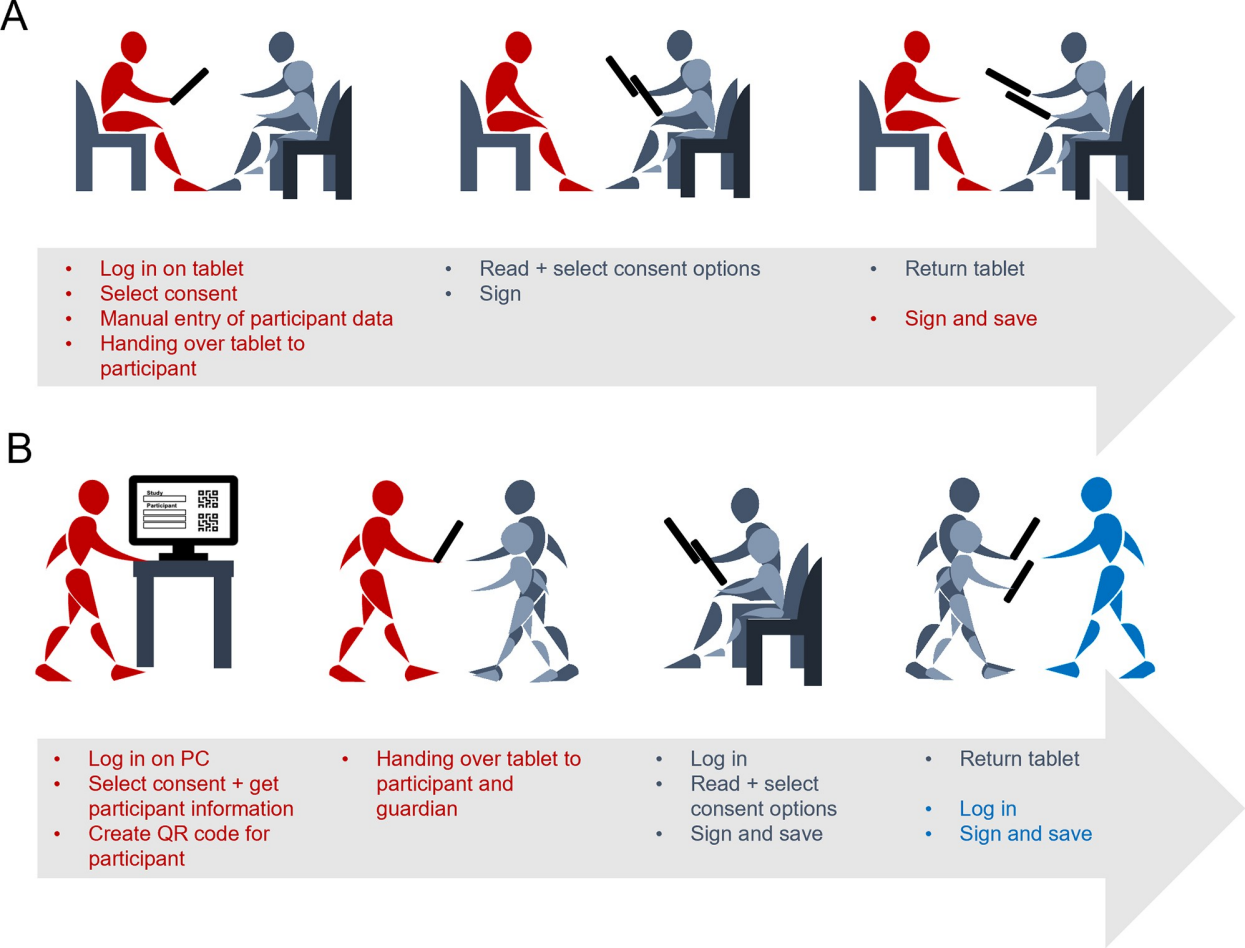
Schematic representation of the informed consent process in pediatrics. The consent process for studies in pediatrics involves the child’s parents or legal representative, as well as the child’s capacity to consent. If the children are old enough, they can give informed consent themselves. Both the guardians and the children are informed about the study protocol, objectives, risks, and potential benefits of participation. (A) Process for obtaining consents using gICS: For each consent, the individual providing information accesses the gICS on the tablet and selects the appropriate consent template. Participant-specific details are then manually inserted. The tablet is then passed to the participant and/or legal representative to review and select the consent content before providing their signature. The participant and/or their legal representative read the consent points, make their selection, sign, and return the tablet to the individual providing information. The individual then finalizes the consent process with their signature. (B) Streamlined process using an additional frontend: The individual providing information logs into the frontend on their personal computer and chooses the appropriate consent templates. Additionally, subject-specific information is obtained through the FHIR server, and a unique QR code is generated for each consent template. Up to four QR codes can be generated simultaneously. These codes are scanned by the child or legal representative, and the corresponding consent template is displayed after logging in. The child or legal representative then selects consent options, signs, and saves the consent, always having the opportunity to clarify specific parts of the consent. The individual providing information will then log onto the tablet, review and sign the consent form. Once signed, the consent form will be transferred to gICS.

gICS serves a dual function by enabling both the administration and acquisition of consents. However, the system design does not allow for temporary storage after the participant has given their consent. Therefore, both the participant and the informing party must sign the document before submitting it. Additionally, participants can complete the consent process independently if the informant becomes inattentive or distracted. As a result, obtaining consent is only possible in direct engagement scenarios. Dealing with lengthy consent documents or multiple consents can be particularly time-consuming, as the person providing information must be present while the participant reads each section of the consent. Additionally, the software lacks the ability to prepopulate fields in electronic consents with participant-specific information, which requires repetitive data entry for multiple consents and can lead to potential errors, further increasing the time required to complete the consent. Fig 4A demonstrates the process for taking consents using gICS without any adaptations. To simplify this process (Fig 4B), an additional frontend has been added to the setup. The gICS integrated FHIR interface enables retrieval of consent templates. Similarly, participant-specific information can be accessed through a FHIR server. Using these options, the new frontend was used to create subject-specific consent templates, which can be accessed using a sample-specific QR code. After completing the consent form, the participant can temporarily save it and then it can be finalized by the person providing the information.

### Captured consents

**Fig 5:**
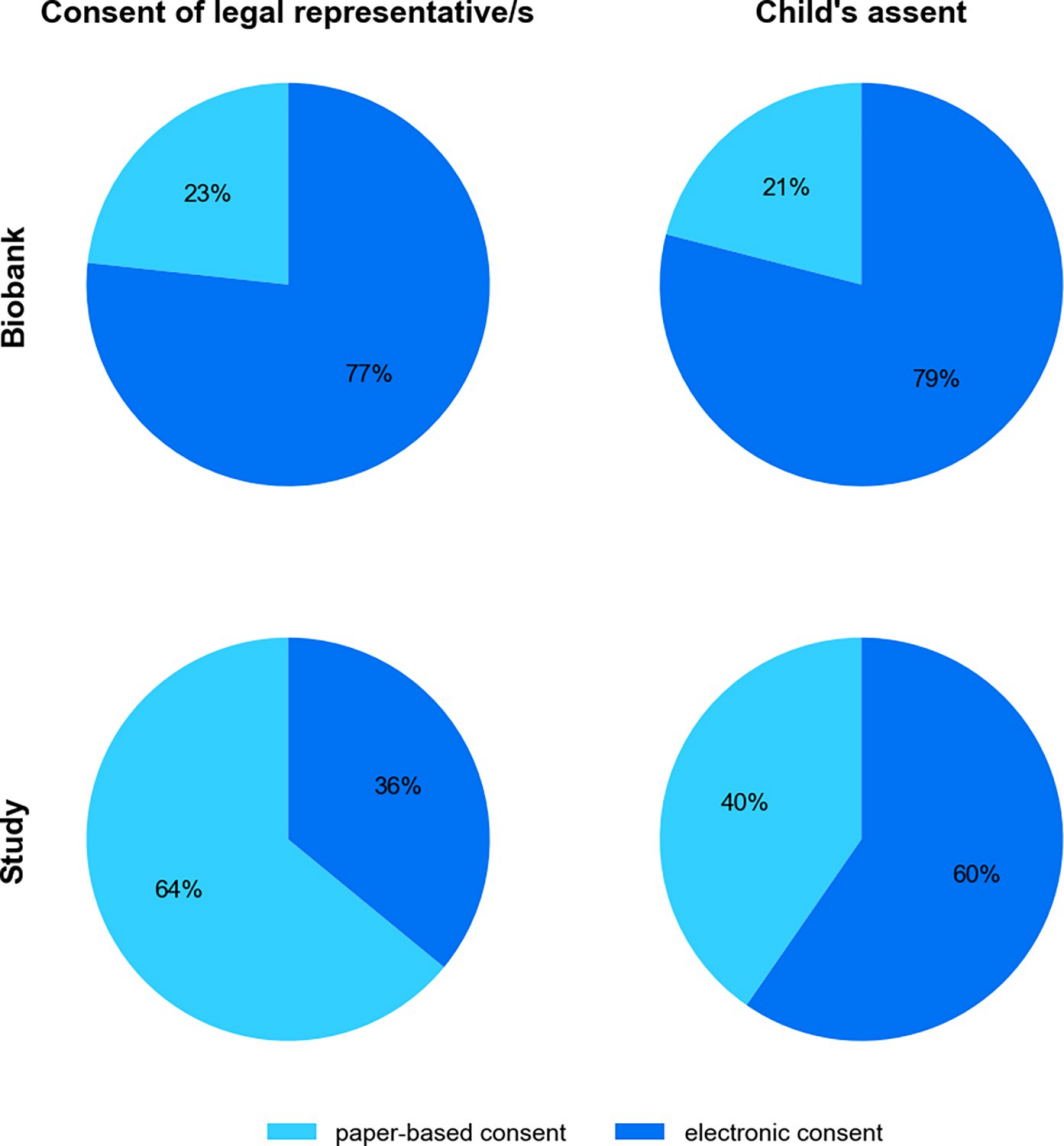
Distribution of captured consents. The distribution of all electronic and paper-based consents collected during the 54-week period is displayed, divided by study and biobank participation.

Recording of consents in electronic format was initiated as a part of the biobank at the pediatric clinic. After the first 9 weeks consent for the study was captured electronically additionally. The offer to complete the consent in paper-based form on-site was not taken up by any of the guardians or children. During the first 9 weeks 140 children were enrolled in the study. Of these, 54.3% (76/140) agreed to participate in the biobank. Approximately two thirds of these participants consented completely electronically to the biobank. In the following 45 weeks both, study and biobank consents, could be captured electronically. 921 additional children and adolescents were enrolled. 29.5% (272/921) of them agreed to partake in the biobank. A total of 481 participants of them approved at least one electronic consent. In 17.0% (157/921) of subjects, authorization was partially obtained electronically and 35.1% (324/921) of them entirely granted their approval electronically. Over the entire period of 54 weeks observed, a total of 1121 consents were recorded electronically, including 598 consents from legal representatives and 523 assents from children. Fig 5 shows the distribution of electronically and paper-based consents obtained for the biobank and study over time.

### Error susceptibility

A comparison was made between the error susceptibility of paper-based and electronically performed consents. A total of 2066 collected consents were checked for errors, including all consents recorded for the biobank throughout the entire period and those recorded for the study from week 10 onwards. The purpose was to determine if there were any differences in the number of incorrect entries. Table 1 displays the frequency of errors in 945 paper-based and 1121 electronically collected consents. Approximately 90.9% of all collected consents were error-free, while 9.1% contained at least one error. Of the electronically captured consents, 99.0% were error-free, compared to 81.3% of paper-based ones. On average, paper-based documents had 0.22 errors per document, compared to 0.01 errors per electronic document. The difference in error rates was statistically significant (p < 0.001). The most frequent errors, found in 159 out of 176 erroneous paper-based documents, were caused by missing signature dates of the participants and/or the person responsible for recording consent. Errors in electronically collected consents were mainly caused by spelling mistakes in names and incorrect filling of free text fields (Table 2). However, all consents, whether paper-based or electronic, were complete with all signatures. Additionally, in all but two consents, components were accurately selected. Overall, calculation of the relative decrease in errors demonstrated a 94.7% (95% CI: 89.43%, 99.87%) improvement with electronic consent recordings compared to paper-based consent for all consents, with of a reduction of 97.31% (95% CI: 97.26%, 97.37%) for consents of children and 92.0% (95% CI: 91.96%, 92.01%) from legal guardians.

**Table 1:** Assessing error susceptibility.

		Electronic consent		Paper-based consent	
		Informed consent of legal representative/s	Child’s assent	Informed consent of legal representative/s	Child’s assent
**Biobank**					
Number of Documents					
	No error	263 (98.5%)	190 (99.0%)	67 (82.7%)	38 (74.5%)
	One error	4 (1.5%)	2 (1.0%)	14 (17.3%)	13 (25.5%)
	Two errors	0 (0.0%)	0 (0.0%)	0 (0.0%)	0 (0.0%)
	Three or more errors	0 (0.0%)	0 (0.0%)	0 (0.0%)	0 (0.0%)
Average number of errors per document		0.01	0.01	0.17	0.25
**Study**					
Number of documents					
	No error	328 (99.1%)	329 (99.4%)	506 (85.8%)	158 (70.9%)
	One error	3 (0.9%)	2 (0.6%)	82 (13.9%)	64 (28.7%)
	Two errors	0 (0.0%)	0 (0.0%)	2 (0.3%)	1 (0.4%)
	Three or more errors	0 (0.0%)	0 (0.0%)	0 (0.0%)	0 (0.0%)
Average number of errors per document		0.01	0.01	0.15	0.30

**Table 2:** Error rates in erroneous paper-based and electronic consents.

Type of error	Paper-based consent	Electronic consent
Missing signer date of participant and/or research staff	159	0
Additional text on the consent form	9	0
Crossing out paragraphs on the consent form	1	0
Missing selection of consent options	2	0
Incomplete identification information	3	0
Spelling mistakes in name	0	8
Incorrectly filing in information in the form	5	3
Missing signature of participant and/or research staff	0	0

## Discussion

The aim of this study was to evaluate the effectiveness of gICS, a digital platform for documenting consent in research, and its integration into the current infrastructure in a pediatric setting. Additionally, the study aimed to assess the advantages of electronic informed consent in pediatrics. The software allowed for the management of versioned consent templates and the electronic recording of consents. Over a period of 54 weeks, 1061 participants were enrolled, and 1121 electronic consents were collected.

A recent investigation revealed a lack of research in the field of pediatrics regarding the application of a digital tool to document consent for research. Although Chen et al. discussed the use of REDCap, they did not evaluate minor assent in addition to the consent of legal representatives [19]. Most published articles focus on providing digital information about medical procedures to improve understanding of the intervention, rather than the consent process itself [10]. Our study found that both parents and children gave positive evaluations of the electronic consent tool. No concerns were raised, which is consistent with the literature [19,20]. However, the biobank study recorded a higher number of electronic consents compared to the regular study. This was due to the distribution of documents in advance to both study subjects and their legal guardians. It is important to note that biobank consent is not mandatory for participation in the regular study, and primarily prefilled forms for the study were brought to appointments. The higher consent rate for the study may be due to the provision of clear and objective information, while the biobank consent form lacks a specific study objective. Personal interaction facilitated a higher consent rate for the biobank, and custodians were subsequently able to provide electronic consent after their queries were addressed. Research on electronic consent collection for HPV vaccination in adolescents found a lower proportion of electronic consent compared to paper-based consent. Organizational factors were suspected as the cause [20], which cannot be ruled out in our setting with certainty.

Obtaining error-free documents is essential for the success of a study. Our analysis shows that using a digital tool can improve the accuracy of completed consent forms. We observed a significant decrease in error rate when obtaining electronic consent forms compared to alternative methods, as reported by Reeves et al. [7]. Including the date of signatures is a simple measure to avoid most errors. In addition to the reduction of errors, electronic consent also simplifies the storage, retrieval, and sharing of documents, thereby reducing physical storage needs and the risk of document loss or damage [21,22]. Despite these benefits, one limitation was observed. Technical issues such as system failures or poor internet connectivity can hinder the use of electronic consent, thereby rendering paper-based consents a necessary fallback.

While the overall findings of the present study indicate that the utilization of a electronic consent process may have a favorable impact, comparable to that observed in previous research on such processes [7,20,22], it is essential to acknowledge and address several limitations in subsequent investigations. It is possible that a selection bias may have been introduced due to the preselection of participants. This may have occurred because families who are more familiar with medical research and possess higher levels of health literacy may have been more likely to take part. The decision-making process for parents granting consent for their child’s involvement in clinical research is influenced by several determinants. Of particular importance are the child’s health status, favorable perceptions of the research team, altruistic motivations and socioeconomic factors [23–25]. These factors were not collected in the study. Despite efforts to recruit families with children across all pediatric subdisciplines, it is possible that these factors may have influenced the results. Additionally, the sample size may not adequately represent the full diversity of the pediatric population. These limitations should be carefully evaluated in future research to ensure more comprehensive and generalizable findings.

Automatically transferring consent information to study software can reduce documentation and recording errors. The software’s container-based architecture allows for a diverse range of integration customization options. The transfer was quickly resolved by using microservices, which enabled the automatic transfer of consent content and storage of consent documents in the archive. This software development approach allows for easy integration into various environments and can be customized to meet specific needs [26,27]. Although multi-client capability was not initially available, it was easily enabled through project-specific access setup. Nevertheless, it is essential to weigh the advantages and drawbacks of this setup. While it offers several benefits, such as improved isolation and scalability, it also raises important considerations regarding multi-tenancy and separate applications for each project. On the one hand, having separate applications can provide a high degree of control and data security and can simplify the management of different projects, as each application is self-contained and does not impact other projects [28]. On the other hand, having multiple applications can lead to increased complexity, higher resource utilization and potential versioning issues, as each application needs to be maintained and updated separately. It is recommended to further evaluate the functionality of project-specific access from version 2023.1.0 onwards as it may help reduce resource usage.

gICS was found to be suitable for its intended purposes. The transition from traditional paper-based consent to digital recording was easily achievable on site. However, currently, consent can only be provided through the application after logging in by the individual providing information and in direct contact due to the inability to save the consent in between. To address this limitation, the software provides the option to obtain consent templates through a FHIR interface. The application has potential for further optimization, particularly in pediatrics where multiple consent forms must be regularly completed with the same identification information. Using prefilled electronic forms for identification with names or study numbers can further reduce sources of error. We have developed a new frontend that includes a customized QR code image. This code allows participants to access a prefilled consent form with all the necessary information. Each participant will receive a unique QR code that provides quick and easy access to their personal form. After scanning the QR code, the participant will be redirected to a page where they can select consent options after logging in. Users can complete, review, and confirm their consent using this method. This approach not only increases efficiency but also provides a user-friendly experience for electronic consent.
